# Candidate Domestication-Related Genes Revealed by Expression Quantitative Trait Loci Mapping of Narrow-Leafed Lupin (*Lupinus angustifolius* L.)

**DOI:** 10.3390/ijms20225670

**Published:** 2019-11-12

**Authors:** Piotr Plewiński, Michał Książkiewicz, Sandra Rychel-Bielska, Elżbieta Rudy, Bogdan Wolko

**Affiliations:** Department of Genomics, Institute of Plant Genetics, Polish Academy of Sciences, 60-479 Poznan, Poland; pple@igr.poznan.pl (P.P.); sryc@igr.poznan.pl (S.R.-B.); Elarudy@op.pl (E.R.); bwol@igr.poznan.pl (B.W.)

**Keywords:** vernalization responsiveness, alkaloid content, pod shattering, gene expression, quantitative trait loci

## Abstract

The last century has witnessed rapid domestication of the narrow-leafed lupin (*Lupinus angustifolius* L.) as a grain legume crop, exploiting discovered alleles conferring low-alkaloid content (*iucundus*), vernalization independence (*Ku* and *Julius*), and reduced pod shattering (*lentus* and *tardus*). In this study, a *L. angustifolius* mapping population was subjected to massive analysis of cDNA ends (MACE). The MACE yielded 4185 single nucleotide polymorphism (SNP) markers for linkage map improvement and 30,595 transcriptomic profiles for expression quantitative trait loci (eQTL) mapping. The eQTL highlighted a high number of *cis*- and *trans*-regulated alkaloid biosynthesis genes with gene expression orchestrated by a regulatory agent localized at *iucundus* locus, supporting the concept that *ETHYLENE RESPONSIVE TRANSCRIPTION FACTOR RAP2-7* may control low-alkaloid phenotype. The analysis of *Ku* shed light on the vernalization response via *FLOWERING LOCUS T* and *FD* regulon in *L. angustifolius*, providing transcriptomic evidence for the contribution of several genes acting in C-repeat binding factor (*CBF*) cold responsiveness and in UDP-glycosyltransferases pathways. Research on *lentus* selected a *DUF1218* domain protein as a candidate gene controlling the orientation of the sclerified endocarp and a homolog of *DETOXIFICATION14* for purplish hue of young pods. An *ABCG* transporter was identified as a hypothetical contributor to sclerenchyma fortification underlying *tardus* phenotype.

## 1. Introduction

The narrow-leafed lupin, *Lupinus angustifolius* L., is a grain legume crop, appreciated as an organic fertilizer that improves soil structure and productivity, as well as providing a source of protein for human and animals. This species has witnessed rapid domestication during the last century. Several important agronomic traits have been identified and transferred into improved germplasm [1]. These traits include, among others, vernalization independence (overlapping loci *Ku* and *Julius*), low-alkaloid content (*iucundus*), reduced pod shattering (*tardus* and *lentus*), soft seededness (*mollis*), white flower color (*leucospermus*) and anthracnose resistance (*Lanr1*).

Vernalization responsiveness is the natural adaptation to climatic conditions, based on the requirement of a prolonged low temperature period during germination to induce flowering [2,3]. Natural dominant mutation in the so-called *Ku* or *Julius* loci diminished the need of vernalization and enabled temperature-independent sowing of *L. angustifolius* [4].

A high level of quinolizidine alkaloids is a typical feature of primitive populations in many lupin species, as these chemical compounds protect plants from pests and fungi [5], however, alkaloids are major antinutritional factors and provide bitter taste [6,7]. Three unlinked low-alkaloid recessive alleles were identified in *L. angustifolius*, and one of them, *iucundus*, was extensively implemented in breeding [8,9]. Some germplasm resources having less than 0.01% of grain alkaloid have been developed [10,11].

Shattering of dry pods is natural process of seed dispersal, however, it is a very undesired trait in modern agriculture because it dramatically decreases harvested yield. Two unlinked recessive alleles contribute to reduced pod shattering in *L. angustifolius*, namely *tardus*, affecting sclerenchyma strips of the dorsal and ventral pod seams, and *lentus*, modifying the orientation of the sclerified endocarp of the pod [12,13].

*L. angustifolius* is natively adapted to the Mediterranean climate which has hot dry summers, because of one of its survival strategies which is impermeability of seed coat to water. Hard-seeded germplasm has a long dormancy period and irregular germination. Recessive soft-seediness allele *mollis* confers water permeability and efficient seed germination [14]. It is the most difficult domestication *L. angustifolius* allele for breeding because the desired phenotype is maternally determined [15]. 

The agronomic potential of *L. angustifolius* has been reduced by high susceptibility to anthracnose, caused by the pathogenic fungus, *Colletotrichum lupini* (Bondar) Nirenberg, Feiler and Hagedorn [16]. The resistance to anthracnose in *L. angustifolius* was revealed to be controlled by several single dominant genes that were discovered in different germplasm resources, namely, *Lanr1* in cultivar Tanjil, *AnMan* in cv. Mandelup, and *LanrBo* in the breeding line Bo7212 [17,18,19].

Several genes contribute to *L. angustifolius* seed and flower color. The most widely exploited is the recessive allele *leucospermus*, affecting anthocyanin synthesis and resulting in bright seeds and white flowers [1]. 

To generate numerous molecular markers for agronomic trait selection in narrow-leafed lupin breeding programs, microsatellite-anchored fragment length polymorphisms (MFLP) fingerprinting has been exploited [20,21]. Trait-associated markers have been developed for *iucundus* (marker iucLi) [22], *Ku* (KuHM1) [23], *mollis* (MoLi) [15], *lentus* (LeM1, LeM2 and LeLi) [13,24], and *tardus* (TaLi, TaM1 and TaM2) [25,26]. Narrow-leafed lupin genomic studies have been greatly facilitated by the incremental development of linkage map carrying sequence-defined markers [27,28,29,30], construction of nuclear genome bacterial artificial chromosome (BAC) libraries [31,32], and assembly of the draft genome sequence [30,33,34].

Recently, a new method of transcriptome-based genotyping-by-sequencing, called massive analysis of cDNA ends (MACE), has been developed [35]. The MACE provides markers anchored in 3′-ends of transcribed sequences, and therefore is directly matching active RNA content of the genome. First implementations highlighted the relevance of the MACE for sequence polymorphism detection, gene expression quantification, transcript-based marker development, and candidate gene identification [36,37,38,39,40,41]. In this study, the MACE protocol was used for development of polymorphic gene-based markers and for quantification of gene expression in mapping population of *L. angustifolius*. These new data were exploited for construction of a linkage map and for determination of expression quantitative trait loci (eQTLs) related to selected domestication traits.

## 2. Results and Discussion

### 2.1. Development of New Polymorphic Markers

The MACE protocol was applied for 89 RILs and for parental lines of *L. angustifolius* mapping population (83A:476 × P27255), yielding 11,864 markers. A total of 9304 markers were localized within gene sequences whereas 2560 markers were found in loci lacking any annotation. There were 4185 MACE markers retained after application of total missing data threshold (counting heterozygotes and no data scores), followed by inference of consensus segregation for genes represented by several single nucleotide polymorphism, SNPs. There were 3532 genes represented by single markers, four genes were represented by pairs of markers with heterogeneous segregation patterns, and 645 markers were localized in unannotated loci. The annotation of markers is provided in the Table S1. 

The MACE is a method providing sequences anchored in the 3′-ends of mRNA and can be used to develop sequence-defined markers, as well as to quantify gene expression [35,36]. In this study both applications of the MACE protocol were exploited, providing molecular markers and gene expression scores related to the same RNA isolates. The MACE marker set was supplemented with 10 newly developed BAC-end derived PCR markers, namely five dCAPS (019A15_3, 026O16_3, 034M08_5, 043N19_3, and 103O20_3), four CAPS (061O23_3, 085K20_5, 085L14_3, and 128I22_5), and one allele-specific PCR marker (085L14_5). The BAC-end based marker allele sequences were deposited in NCBI Genbank under accession numbers (MN518055-MN518073). Information on primer pair sequences, PCR primer annealing temperature, PCR product lengths, enzyme used for polymorphism detection, and restriction product lengths for both alleles is provided in Table S2. For the past 15 years, the use of BAC-derived PCR markers has been a method of choice in studies involving *L. angustifolius* genome physical and linkage mapping. Because the *L. angustifolius* karyotype carries numerous small and very uniform chromosomes, the BAC-derived markers have been frequently used as chromosome-specific landmarks to validate physical linkage of particular genome regions, as well as to facilitate assignment of particular chromosomes to linkage groups [31,42,43,44,45,46,47]. The BAC-derived markers have also been exploited for fine mapping of a region carrying a candidate gene for vernalization independence *Ku* locus, as well as for comparative mapping of genes from isoflavonoid and fatty acid synthesis pathways [48,49,50,51]. In this study, BAC-derived markers were developed to localize on the linkage map some clones identified during our previous studies and confirmed to carry repetitive elements.

### 2.2. Construction of a Linkage Map

Markers used for linkage map development included 4,185 MACE and 10 BAC-end PCR markers developed in this study as well as previously published data including seven trait loci (*Ku*, *tardus*, *lentus*, *mollis*, *leucospermus*, *iucundus* and *Lanr1*) [21], eight trait related markers (TaM1, TaM2, LeM1, LeM2, KuHM1, AntjM2, MoA, MoLi) [13,15,24,25,52,53], and 109 BAC-derived markers anchoring particular linkage groups to chromosomes [43,44,45,46,47,49,50,51,54]. The segregation data for markers used for linkage mapping and the calculated χ^2^ *p*-values of distortion from expected 1:1 segregation are provided in Table S3. There were 4309 markers localized on the genetic map, which constituted 20 linkage groups, carrying from 144 to 304 markers (215 on average) and 16 markers remained unmapped (Table 1). 

**Figure 1:**
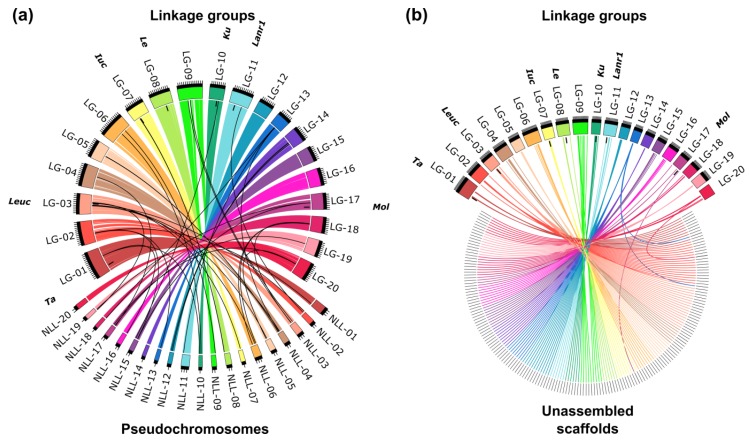
Collinearity links matching narrow-leafed lupin linkage groups (LG-01–LG-20) and: (**a**) pseudochromosomes (NLL-01–NLL-20) and (**b**) unassembled scaffolds. Ribbons symbolize homologous links identified by DNA sequence similarity. Chromosomes and linkage groups are drawn to scale indicated by ticks (10 Mbp and 10 cM). Postions of the following major domestication loci are indicated: *Tardus* (*Ta*), *leucospermus* (*Leuc*), *iucundus* (*Iuc*), *lentus* (*Le*), *Ku*, *Lanr1,* and *mollis* (*Mol*).

The segregation pattern of 59.7% of the markers was redundant, and therefore the map contains 1735 loci, namely from 60 to 120 loci per linkage group. The lengths of linkage groups vary from 78.5 to 156.38 cM, reaching 2163.63 cM in total. The results of linkage mapping are provided in Table S4. A high percentage of markers matching particular linkage groups with corresponding pseudochromosomes, reaching from 96.6% (chromosome NLL-16) to 100% (chromosomes NLL-14 and NLL-20), highlighted the collinearity between published *L. angustifolius* draft genome sequence [34] and this version of linkage map (Figure 1a). Major issues were found for chromosomes NLL-16 (block of eight adjacent markers representing ~300 kbp localized in the linkage group NLL-01), NLL-15 (block of six adjacent markers covering ~200 kbp mapped in the linkage group NLL-17), and NLL-12 (five markers mapped in the linkage group NLL-14). As many as 209 unassembled scaffolds were localized on the genetic map, namely from six to 22 scaffold per linkage group (Figure 1b). A comparison of the number of markers assigned to particular chromosomes, scaffolds, and linkage groups is provided in Table S5.

Mapping data from the most recent *L. angustifolius* linkage maps [30,34] were not incorporated to our map due to a limited number of RIL lines common for all three studies (about 70), as well as due to observed inconsistency in segregation patterns between physically linked markers originating from different studies, indicating diverse genetic origin of some RILs having the same numbers assigned, putatively resulting from seed admixture or cross-pollination during seed multiplication. Seeds of the mapping population were shared between the Department of Agriculture and Food Western Australia and the Institute of Plant Genetics, Polish Academy of Sciences, in the year 2003, and maintained independently thereafter. As lupin breeding was recently licensed to the private sector in Australia it may be currently impossible to access original set of RILs developed for this mapping population. Similar issues with possible cross-pollination during mapping population development have also been reported for 43 RILs from the recently published linkage map of yellow lupin, *L. luteus* [55], as well as for one RIL in white lupin, *L. albus* [56]. Nevertheless, the total number of RILs used in the most recent *L. angustifolius* genome mapping study, namely 87 lines with only 78 lines overlapping with previous mapping studies, was too low to provide the high resolution required for significant improvement of genome assembly, and resulted in high marker redundancy, reaching 89.9% [30]. 

### 2.3. Gene Expression Profiling, Gene Ontology Enrichment, and Expression Quantitative Trait Loci Mapping

The MACE analysis provided normalized gene expression levels for all RILs analyzed. Namely, 30,595 genes revealed nonzero expression for at least 1 RIL, 25,024 genes for at least 30% of RILs, 23,557 genes for at least 50% of RILs, and 15,686 genes for all RILs. The normalized gene expression values for mapping population and parental lines (83A:476 and P27255) are provided in Table S6. The gene expression patterns in the mapping population were associated with domestication trait segregation (wild alleles used as positive values). Genes with a statistically significant association (FDR p-value threshold of 0.01) were identified for all domestication traits analyzed, namely 98 genes for *iucundus*, 50 for *Ku*, 35 for *leucospermus*, 29 for *lentus*, 17 for *tardus*, 11 for *Lanr1* and five for *mollis*. The values of the *t*-Student test association between domestication trait segregation and gene expression patterns, including FDR correction and statistical significance analysis, are provided in Table S7. The gene ontology (GO) enrichment analysis of genes with expression pattern associated with *iucundus* trait segregation highlighted lysine biosynthesis and lysine metabolism, as well as cofactor binding and coenzyme binding, as the most overrepresented processes and functions, respectively (Table S8). This was an expected outcome as quinolizidine alkaloids are derived from lysine via a series of chemical reactions [57]. GO analysis for *tardus*-associated genes revealed iron-sulfur cluster assembly, metallo-sulfur cluster assembly, and cofactor biosynthesis process enrichments. No statistically significant GO enrichments were identified for genes associated with *Ku*, *leucospermus*, *lentus*, *mollis* and *Lanr1* traits.

Composite interval mapping revealed the presence of numerous eQTL peaks close to domestication trait loci. Within a genetic linkage distance of 2 cM from a particular domestication trait locus, from one (*mollis*) to 61 (*iucundus*) genes had eQTL peaks localized (Table 2). The LOD values for eQTL permutation test are provided in Table S9, whereas data on eQTL localization on the linkage map are provided in Table S10. Some potential candidate genes were identified for all analyzed loci, except anthracnose resistance locus *Lanr1*. As plants were not inoculated to allow long-range gene profiling during their development, including the generative phase, genes related to anthracnose were putatively not activated in the experiment. Anthracnose resistance will be addressed in another study. Here, genes identified for *iucundus*, *Ku*, *lentus*, *tardus*, *mollis* and *leucospermus* are discussed.

### 2.4. Genes Identified for Low-Alkaloid Iucundus Locus

The high number of genes revealed for *iucundus* locus might be related to the complexity of the alkaloid biosynthesis process and the number of genes involved. Taking into consideration the position of gene coding sequences in the genome, only a relatively small fraction of eQTL genes revealed for *iucundus* (18%) was *cis*-regulated, whereas the vast majority (74%) was *trans*-regulated. Furthermore, from 34 genes for *iucundus* that had association values between their expression patterns and trait segregation above 0.5 or below −0.5, as many as 30 revealed a positive association with wild, high alkaloid phenotype. Moreover, as many as 16 genes highly associated with *iucundus* revealed to have their major eQTL locus explaining more than 50% of their observed expression variance localized directly at *iucundus,* or very close to it (Table 3). Many of these genes are hypothesized to be involved in alkaloid biosynthesis process. Such an observation strongly supports a hypothesis that *iucundus* locus in *L. angustifolius* encodes a single regulatory agent controlling this complex secondary metabolic pathway and differentiating between high and low quinolizidine alkaloid biosynthesis profiles.

**Figure 2:**
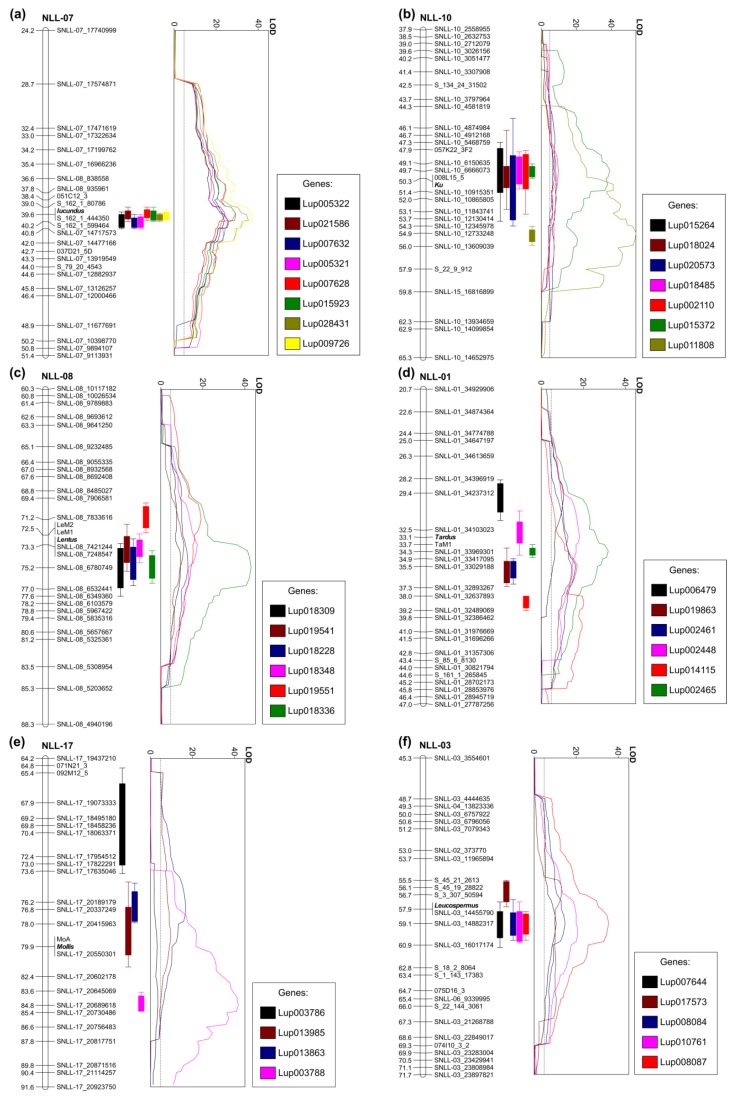
Major expression quantitative trait loci (eQTLs) revealed for narrow-leafed lupin domestication trait loci: (**a**) main alkaloid content *iucundus* locus, (**b**) vernalization responsiveness *Ku* locus, (**c**) pod shattering *lentus* locus, (**d**) pod shattering *tardus* locus, (**e**) soft seededness *mollis* locus, and (**f**) white flower color *leucospermus* locus. Linear plots show LOD values (threshold 4.8), whereas vertical bar graphs visualize eQTL ranges (outer, LOD_max_-2 and inner, LOD_max_-1) on corresponding linkage group fragments. Linkage groups are drawn to scale indicated by ticks and labels.

Among the genes with expression positively associated with high alkaloid phenotypes in the RIL population, the highest LOD values of eQTLs were revealed for Lup009726 (OIV96299.1, LOD 37.3), Lup028431 (OIV96574.1, LOD 35.4), Lup015923 (OIW10551.1, LOD 29.4), and Lup007628 (OIV89004.1, LOD 29.2) (Figure 2a). The Lup009726 product revealed 99.5% sequence identity to lysine/ornithine decarboxylase LDC (BAK32797.1) protein which catalyzes the first step of quinolizidine alkaloid biosynthesis [57]. Expression of the *LDC* gene has been confirmed to be associated with alkaloid content in *L. angustifolius* by several independent studies [58,59,60]. Lup028431 encodes purine permease transporter 1 (PUP1), PUP proteins which are generally involved in alkaloid biosynthesis and transport. Nicotine uptake permease from *Nicotiana tabacum* (*NtPUP1*), for example, affects nicotine metabolism, as well as regulates the *ETHYLENE RESPONSE FACTOR 189*, a key transcription factor in nicotine biosynthesis pathway [61,62]. Indeed, Lup028431 was selected in another study as potential *L. angustifolius* quinolizidine alkaloid biosynthetic gene because it revealed similar expression pattern to the *LDC* gene [63]. Lup015923 has been annotated as *MAJOR LATEX PROTEIN 423* (*MLP423*, AT1G24020), which is hypothesized to be involved in stress responsive activation of biosynthetic pathway of coumestrol, a coumestan isoflavone in soybean [64]. Lup015923 was recently highlighted as one of candidate quinolizidine alkaloid biosynthesis genes in *L. angustifolius* due to highly elevated expression in bitter P27255 accession [58]. Lup007628 (OIV89004.1) is the *ETHYLENE RESPONSIVE TRANSCRIPTION FACTOR RAP2-7*, a candidate locus for *iucundus*, evidenced by a gene expression study involving transcriptome sequencing of four accessions and quantitative RT-PCR profiling of 14 accessions differing in alkaloid content, as well as by molecular marker development and linkage mapping [59,60]. Interestingly, closely located to *RAP2.7* at *iucundus* locus, another *cis*-regulated component, *Fe SUPEROXIDE DISMUTASE 2* (Lup007632, OIV89008.1), revealed similarly high LOD and explained eQTL variance values, but opposite direction of association. Other *iucundus*-associated genes have included Lup005321 (OIW13431.1) and Lup005322 (OIW13432.1) encoding homologs of *DIHYDROFLAVONOL 4-REDUCTASE* which is one of the key genes from anthocyanin biosynthesis pathway [65]. The set of genes with highly significant eQTLs localized in the *iucundus* region also includes a Lup021586 (OIV95196.1) gene encoding *HXXXD*-type *ACYL-TRANSFERASE* (*LaAT*, AB581532.1). The expression profile of *LaAT* has been highly associated with alkaloid content in *L. angustifolius* [58,59,60,66]. Moreover, one of the homologs of this gene, LAGI01_35805, has been recently designated as a candidate gene underlying low-alkaloid *pauper* locus in *L. albus*, as evidenced by linkage mapping and validation survey in a set of 127 bitter and 23 sweet accessions [56].

In addition to Lup007628 and Lup007632, nine other *cis*-regulated genes revealed eQTLs localized at *iucundus* locus, including three hypothetical components of alkaloid biosynthesis pathways, Lup007706 (OIW07664.1), Lup017658 (OIW07643.1), and Lup032669 (OIW21355.1). Lup007706 encodes a representative of a *S*-adenosyl-l-methionine-dependent methyltransferases superfamily protein. N-methylation of quinolizidine alkaloids was confirmed to occur in crude protein extracts from *Laburnum anagyroides* carrying *S*-adenosyl-l-methionine: cytisine N-methyltransferase [67]. Moreover, a homolog of *S*-adenosyl-l-methionine-dependent N methyltransferase catalyzes a nitrogen methylation involved in vindoline alkaloid biosynthesis in Madagascar periwinkle (*Catharanthus roseus*) [68]. Lup017658 encodes a *4-HYDROXY-TETRAHYDRODIPICOLINATE SYNTHASE* gene which is generally involved in biosynthesis of l-lysine, a precursor of quinolizidine alkaloids. This gene revealed considerably elevated expression in bitter accessions of *L. angustifolius*, indicating its hypothetical involvement in alkaloid biosynthesis pathway [59]. Lup032669 encodes the *CARBOXYLESTERASE 1* gene. *CARBOXYLESTERASE 1* was evidenced to be involved in one of the final three steps of noscapine alkaloid biosynthesis [69]. To summarize, this study highlighted a relatively high number of alkaloid biosynthesis genes with gene expression orchestrated by a regulatory agent(s) localized at *iucundus* locus. This study provided novel evidences supporting the concept that *RAP2.7* may control low-alkaloid *iucundus* phenotype, however, further evidence would require *cis*-trans tests. Nonetheless, such studies are hampered by very low transformation efficiency in narrow-leafed lupin [70].

### 2.5. Genes Revealed for Vernalization Independence Ku Locus

The P27255 parent is late flowering and requires vernalization for flowering induction, whereas the 83A:476 parent is early flowering and vernalization independent. In this study, seeds were subjected to vernalization procedure to ensure transition from vegetative to generative phase in all analyzed RILs. Such an approach could result in diminishing of some differences in expression profiles of vernalization-responsive genes between early and late flowering RILs. However, despite this partial pre-sowing vernalization, relatively a large number of genes revealed to have their eQTL peaks closely localized to *Ku* locus (Table 4, Figure 2b). Contrary to *iucundus*, the same ratio of *cis*- and *trans*-regulation for major eQTL loci was observed (44%). Genes showing the highest gene expression association and eQTL peak co-localization (≤2 cM) with *Ku* included Lup011808 (OIW03171.1, LOD 41.0) and Lup015372 (OIW20567.1, LOD 38.9). Lup015372 encodes hypothetical uncharacterized protein, whereas Lup011808 a homolog of *A. thaliana CALCIUM/CALMODULIN-REGULATED RECEPTOR-LIKE KINASE 1*, *CRLK1*. *CRLK1* confers cold responsiveness in plants via C-repeat binding factors (*CBF*) pathway [71]. Overexpression of the *CBF* in *Arabidopsis* delays flowering by promoting the expression of *FLOWERING LOCUS C* (*FLC*), indicating a link between cold signaling and flowering time regulation [72]. One of the downstream genes in this pathway is *INDUCER OF CBF EXPRESSION 1* (*ICE1*) which integrates cold signals into *FLC*-mediated flowering pathway [73]. Another cross-talk between cold response and flowering initiation pathways, involving a *SUPPRESSOR OF OVEREXPRESSION OF CONSTANS 1* (*SOC1*) gene was also identified [74]. A recent study confirmed that *CBF* pathway affects flowering time but does not affect vernalization response in *Arabidopsis* [75]. Moreover, a calcium and calmodulin-binding protein kinase (*NtCBK1*) from *N. tabacum* functions as a negative regulator of flowering; high levels of *NtCBK1* in the shoot apical meristem extended the vegetative phase of growth [76]. Indeed, *CRLK1,* in this study, was revealed to be positively associated with late flowering phenotype. These observations bring attention to the hypothetical involvement of calcium and calmodulin link with *FLC* pathway in flowering time regulation in *L. angustifolius*. Legume genomes do not contain *FLC* homologs but other genes from this pathway, including activators and repressors of *FLC*, are present [77].

Other *Ku* eQTLs include Lup011781 (OIW03144.1), Lup011739 (OIV96743.1), Lup011836 (OIW03199.1), and Lup002110 (OIW20134.1) sequences. Lup011781 has been identified as a homolog of *MHM17-10* (AT5G56980) gene. It is pathogen-associated molecular pattern-induced gene with unknown function, putatively participating in jasmonic acid pathway [78]. Lup011739 encodes a homolog of *GALACTURONOSYLTRANSFERASE-LIKE 10*, which is involved in cell wall organization and its expression is regulated by *FLAVIN-BINDING KELCH REPEAT, F-BOX 1* (*FKF1*), blue light receptor, and well-known photoperiodic flowering time regulator [79]. Lup011836 encodes general transcription factor IIH subunit 2 (*GTF2H2*), performing basic functions in transcription and nucleotide excision repair of damaged DNA. Lup002110 has been annotated as a representative of the UDP-glycosyltransferases protein family. One of the *A. thaliana* UDP-glycosyltransferases, *UGT87A2*, was revealed to be involved in the regulation of flowering in vernalization and gibberellin pathways via the flowering repressor *FLC* [80]. UGT87A2 vs. OIW20134.1 protein alignment revealed 96% coverage, 31% identical sites, and 46% positive sites.

As plants in this study were subjected to a moderate vernalization procedure, differences in gene expression resulting from variation in vernalization responsiveness should be reduced as compared with those expected for nonvernalized plants. This reduction might be highlighted by a relatively low LOD value (as compared with other eQTLs from this region) of the major *L. angustifolius* gene underlying vernalization responsiveness, a homolog of *A. thaliana FLOWERING LOCUS T* (*FT*), *LanFTc1* (Lup015264, OIW03334.1, LOD 7.0) [49,81]. As expected, *LanFTc1* revealed negative association with late flowering phenotype. Similar LOD values were also revealed for Lup018024 (OIV92673.1), Lup020573 (OIW03269.1), and Lup018485 (OIW19675.1) genes. Lup018024 encodes a homolog of bZIP transcription factor, *FD*, which mediates signals from the *FT* gene at the shoot apex and promotes plant flowering in general [82]. However, in this study, *FD* expression revealed a positive association with late flowering phenotype. In *Arabidopsis*, the *FT-FD* complex induces the transcription of several floral-promoting genes, such as *SOC1* and *FRUITFULL* (*FUL*), which accelerate flowering, as well as *APETALA1* (*AP1*) and *LEAFY* (*LFY*), which control floral meristem identity [83]. Indeed, Lup018485 revealed similarity to *AGAMOUS-LIKE 8* (*AGL8*)/ *FUL* and *AP1* MADS-box transcription factors and was found to be negatively associated with late flowering phenotype. Lup020573 was annotated as MYB60 transcription factor. MYB transcription factors perform various regulatory functions in plants in responses to biotic and abiotic stresses, development, differentiation, metabolism, defense, etc. [84].

To summarize, analysis of *Ku* eQTLs shed light on vernalization pathway in *L. angustifolius*, providing transcriptomic evidence for the contribution of several genes acting upstream of *FLC* in *CBF* and *UDP*-glycosyltransferases pathways. The study also revealed transcriptomic contribution of conserved mechanism of *FT-FD* regulon on transition from vegetative to generative development phase in *L. angustifolius*.

### 2.6. Genes Profiled for Lentus and Tardus Pod Shattering Loci 

The recessive *lentus* (*le*) allele changes the orientation of the sclerified endocarp in the pod, substantially reducing torsional forces after drying [12]. In this study, the following three genes highly associated with *lentus* were identified to have major eQTL peaks localized in the proximity of this locus: Lup018336 (OIW06948.1, LOD 43.0), Lup018348 (OIW06960.1, LOD 17.4), and Lup018228 (OIW06846.1, LOD 13.0) (Table 5, Figure 2c). All these genes originated from the same region at chromosome NLL-08, carrying *lentus*. Lup018336 encoded a homolog of *A. thaliana* fiber protein carrying *DUF1218* domain. The genome of *A. thaliana* contained 15 members of *DUF1218* genes. Members of the *DEAL* subfamily of the *DUF1218* confer bilateral symmetry of *Arabidopsis* leaves by controlling proper coordination of cell proliferation between different domains of the leaf lamina margin [85]. Another group of *DUF1218* genes has been related to secondary cell wall biosynthesis and includes *AtUNKA* (At4g27435), *MODIFYING WALL LIGNIN-1,* and *MODIFYING WALL LIGNIN-2* (At1g31720/MWL-1 and At4g19370/MWL-2) [86,87,88]. Lup018348 encodes a homolog of *DETOXIFICATION14*, a member of the multidrug and toxic compound extrusion (*MATE* efflux) family [89]. MATE transporters perform various functions including phytohormone transport, secondary metabolite transport, xenobiotic detoxification, aluminium tolerance, disease resistance, tip growth processes, and senescence [90]. Some MATE proteins have been involved in the transport of anthocyanins or proanthocyanidins to vacuoles and in the flavonoid metabolism pathways [91,92]. Anthocyanins are accumulated in cell vacuoles and are responsible for diverse pigmentation from orange to red, purple, and blue [93]. Interestingly, Lup018336 revealed positive gene expression association with pod shattering phenotype, whereas Lup018348 was positively associated with nonshattering pods. These results are in line with the general observation that *le* allele affects a pod pigmentation, resulting in a purplish hue of young pods and a bright yellowish-brown color on the internal surface of mature pods. Lup018348 may be responsible for this pigmentation, whereas Lup018336 for pod shattering in *L. angustifolius*.

The recessive *tardus* (*ta*) allele affects the sclerenchyma strips of the dorsal and ventral pod seams, greatly increasing the fusion of two pod halves and moderately hampering their separation when drying [12]. Two genes revealed high association and eQTL peak co-localization with *tardus*, namely Lup002465 (OIW17837.1) and Lup002448 (OIW17820.1) (Table 5, Figure 2d). Lup002465 encodes BolA-like family protein with unknown function. Lup002448 is a G family ATP-binding ABC transporter. Such a transporter in rice (*RCN1*) is required for hypodermal suberization of roots [94]. Similarly, *ABCG1* confers suberin formation in potato tuber periderm [95]. Some ABCG transporters are involved in sclerenchyma fiber development via monolignol transport in lignin biosynthesis pathway [96,97]. Moreover, one of ABCG transporters has been revealed to be involved in the silicon-induced formation of Casparian bands in the exodermis of rice [98]. ABCG transporters also perform other diverse functions, including abiotic and biotic stress responses, however, these examples provide non-negligible support to select Lup002448 as a candidate gene involved in *tardus* trait.

### 2.7. Gene Related to the Soft Seededness Mollis Allele

Recessive allele *mollis* provides water permeable testa at maturity [14,99]. Seed dormancy in legumes is related to the deposition of phenolics and, hypothetically, development of suberin-impregnated layers of palisade cells as observed in pea and soybean [100,101]. Only one highly associated gene was revealed by eQTL analysis, Lup013985 (OIW15058.1), annotated as a protein FAM32A/7-dehydrocholesterol reductase (homolog of *A. thaliana DWARF5* gene) (Table 5, Figure 2e). Because the sequence homology of these genes between *L. angustifolius* and *Arabidopsis* is quite low, it is difficult to elucidate a particular function by comparative analysis. It can be concluded that it is putatively a gene involved in plant sterol metabolism. Plant sterols are essential structural components that influence biophysical properties of membranes such as permeability and fluidity [102]. Mutation in one of enzymes contributing to steryl glycoside biosynthesis pathway, UDP-Glc:sterol glycosyltransferase, alters embryonic development, seed suberin accumulation, and cutin formation in the seed coat, resulting in abnormal permeability [103]. Recently, it has been evidenced that a maternally deposited endosperm cuticle underlies this seed coat permeability in *A. thaliana* [104]. *Mollis* is also maternally determined and as such is considered to be the most difficult *L. angustifolius* domestication gene for selection by phenotype observation. Lup013985 cannot be considered to be a candidate gene conferring *mollis* allele, because it is located in different chromosome than *mollis* locus, however, it might be considered to be a hypothetical *trans*-regulated component eventually contributing to *mollis* phenotype.

### 2.8. Genes with eQTL Loci Matching White Flower Color Leucospermus Allele

Recessive *leucospermus* allele confers white flower and bright seed pigmentation in *L. angustifolius*. A similar trait in pea was conferred by a basic-helix-loop-helix (bHLH) transcription factor [105] but eQTL analysis did not highlight any bHLH transcription factor with LOD peak close to *leucospermus* locus. Two genes with expression positively associated with recessive allele revealed eQTL peaks close to *leucospermus*, namely Lup008087 (OIW21684.1) and (Lup017573) OIV97389.1 (Table 5, Figure 2f). Lup008087 encodes ubiquitin-60S ribosomal protein L40 (RPL40A) isoform and is localized in Scaffold_168_4 mapped in this study in linkage group NLL-03 close to *leucospermus*, however, a particular biological function of *RPL40* gene is unknown. Lup017573, from the chromosome NLL-15, revealed similarity to the NRT1 and PTR family proteins. Three eQTLs revealed a negative association with recessive allele, including Lup008084 (OIW15287.1) annotated as F-BOX/WD repeat-containing protein. Interestingly, a single mutation in an F-BOX domain-containing protein, OsFBX310, confers brown hull phenotype in rice resulting from a high content of total flavonoids and anthocyanins [106], however, putatively due to the large evolutionary distance between monocots and dicots, sequence alignment reveled very limited similarity between the OsFBX310 and OIW15287.1 protein sequences.

### 2.9. Applicability of MACE for Gene-Based Studies

This study is the first report on exploitation of the MACE for *L. angustifolius* genome and transcriptome analysis. As a method for gene expression analysis, the MACE was first used in chronic kidney disease survey [35] and in de novo transcriptome analysis of *Calliphora vicina* pupae [37]. The MACE was also exploited for stem rust transcriptomic response in perennial ryegrass (*Lolium perenne*), highlighting a candidate *LpPg1* resistance gene and yielding numerous SNPs which were further transformed into PCR-based molecular markers [36]. The MACE protocol was also applied in pea (*Pisum sativum*) providing single nucleotide variants subsequently converted into CAPS markers [38]. Furthermore, MACE-based studies in pea resulted in the identification of a new mutant allele of the key nodulation gene *Sym33* [107]. The MACE was also used for transcriptomic profiling of *Phaseolus vulgaris* seeds and *Solanum lycopersicum* pollen [39,40]. The MACE was also exploited for GWAS, tagging several candidate genes for salt stress tolerance in *Triticum aestivum* [41].

Several previous *L. angustifolius* genotyping approaches were based on diversity arrays technology (DArT) profiling. DArT studies have highlighted low genetic diversity in narrow-leafed lupin breeding material as compared with primitive and wild germplasm [108]. This domestication bottleneck resulted from narrow genetic variability of exploited resources and significantly limited adaptation range in this crop [109,110]. The DArT-seq has also been exploited for genome-wide association studies (GWAS) targeting several narrow-leafed lupin phenology and yield traits, but it did not provide any candidate gene with significant associations between a marker and a quantitative trait [111,112].

In this study, the MACE was revealed to be an advantageous technique for marker development and gene expression profiling. The eQTL mapping highlighted numerous genes involved in the vernalization response and alkaloid biosynthesis, providing a valuable contribution for further advancement of knowledge on the complexity of molecular networks controlling these two biological processes. Taking into consideration the recent improvements in deciphering the molecular basis underlying early flowering and low-alkaloid phenotypes, as well as addressing results reported here, *L. angustifolius* can serve as a reference model for such studies across the whole genus. Moreover, information about candidate genes identified in *L. angustifolius* can be translated to other legume species as these processes are generally conserved.

### 2.10. Recommendations for Improving Narrow-Leafed Lupin As a Crop

During the process of *L. angustifolius* domestication several agronomic traits were identified and transferred into improved germplasm by classical selection approaches. Current breeding materials and cultivars usually carry desired alleles of all major domestication traits in homozygous state (*Ku, iucundus, lentus, tardus, mollis, leucospermus* and *Lanr1*). However, domestication process was highly focused on these traits and resulted in approximately threefold reduction in genome-wide diversity across domesticated accessions as compared with their wild relatives [112]. Further improvement of this species as a crop will require harnessing of primitive germplasm and subsequent reselection of domesticated alleles in the progenies. One of the most challenging issue is related to the influence of global warming on temperature and rainfall patterns in all major areas where lupins are currently cultivated. This issue could be partially resolved by SNP-based selection of wild accessions of narrow-leafed lupin with well-established local adaptation to warm and dry climate of the eastern Mediterranean basin [111]. Novel opportunities for reducing the time required for transition between phenological phases could also be uncovered by exploitation of natural variability in genes from vernalization and cold pathways highlighted in this study, particularly *LanFTc1*, *CRLK1*, *FD*, *UGT85A2*, *GAUT10*, and *MYB60*. Moreover, a common issue related to dry and warm weather patterns, which are expected to occur more frequently due to changing climate, is pod dehiscence. Identified candidate genes for *lentus* (a homolog carrying DUF1218 domain) and *tardus* (an *ABCG5* transporter) await further genotypic and phenotypic exploration in wide genetic background because mapping population represents only a small fraction of diversity existing in *L. angustifolius* germplasm.

## 3. Materials and Methods

### 3.1. Plant Material

The reference 83A:476 × P27255 recombinant inbred line (RIL) population (*n* = 89, F_8_) of *L. angustifolius* [27] delivered by the Department of Agriculture and Food Western Australia was used in the study. This population was developed from a cross between a domesticated Australian breeding line (83A:476) and a wild accession from Morocco (P27255). The line P27255 is late flowering and vernalization-responsive (recessive allele *ku*), pod shattering (dominant alleles *Tardus* and *Lentus*), hard seeded (*Mollis*), blue flower and dark seed color *Leucospermus*), high alkaloid (*Iucundus*) and anthracnose susceptible (*lanr1*). 83A:476 has an opposite allele combination (*Ku*, *tardus*, *lentus*, *mollis*, *leucospermus*, *iucundus*, *Lanr1*). Both parental lines are homozygous in relation to these alleles.

### 3.2. Controlled Environment Experiment

Seeds of mapping population and parental lines (83A:476 × P27255) were vernalized for 16 days at 4 °C in darkness on Petri dishes with moist filter paper. Filter paper (Chemland, Stargard, Poland) was changed every four days to maintain phytosanitary conditions. Following vernalization, plants were transferred to pots (2 plants per 11 cm × 11 cm pot, about 8 cm between plants) and grown in controlled conditions (photoperiod 16 h, temperature +25 °C day and +18 °C night) at the Wielkopolska Center of Advanced Technologies in Poznań, Poland. Tissue was sampled from young leaves two times a day, 4 h after beginning of photoperiod and 1 h before the end of photoperiod on the 28th, 36th, and 44th day from sowing. Five biological replicates were collected.

### 3.3. Massive Analysis of cDNA Ends

Frozen plant tissue (50 mg, −80 °C) was homogenized using TissueLyser II (Qiagen, Hilden, Germany) and two stainless steel beads (ø 5 mm) placed in a 2 mL tube (Eppendorf, Hamburg, Germany). RNA isolation was performed using SV Total RNA Isolation System (Promega, Madison, WI, USA) according to the protocol. The concentration of RNA was measured using NanoDrop 2000 (ThermoFisher Scientific, Waltham, MA, USA) and A260/A280 ratio. RNA quality was visualized by 1% agarose gel electrophoresis (1X TAE) of denaturated samples. RNA concentration was equalized to 400 ng/µL in nuclease-free water. Samples from particular line (representing 5 terms and 5 biological replicates) were bulked together in equal aliquots. 10 µL of mixture (4 µg of RNA) was subjected to the MACE protocol. The MACE profiling and SNP calling was outsourced (GenXPro, Frankfurt, Germany). The MACE reads were aligned to the *L. angustifolius* genome assembly [34] (http://www.lupinexpress.org). Normalization procedure was as follows [113]: The average raw count of each gene within a library was divided by the geometric mean of all counts in all samples and the median of the quotients was calculated per library. Each raw count was then divided by the library-specific median value.

### 3.4. Molecular Markers and Linkage Mapping

The 83A:476-like scores were assigned as “a”, the P27255-like scores as “b”, and the heterozygotes as “h”. If several cosegregating MACE markers in particular gene were identified, the marker with the lowest percentage of missing data was chosen to infer consensus segregation representing each cluster. To provide a mapping file, heterozygote scores were removed. Accepted missing data threshold was 11%. Chi-square (χ^2^) values for Mendelian segregation were estimated using the expected 1:1 ratio. The calculation of probability was based on χ^2^ and 2 degrees of freedom. Based on the segregation distortion observed in recently published *L. angustifolius* linkage map versions [29,30,34], χ^2^ *p*-value threshold of 1 × 10^−7^ was applied. 

Segregation data for domestication traits and tightly linked SSR-derived markers were included in the study [13,21,22,23,25,52,53]. Moreover, to provide landmarks for chromosome map integration, recently published BAC-derived markers were incorporated [43,44,45,46,47,49,51]. Additionally, novel markers were developed using BAC-end sequences. PCR primers were designed using Primer3′lus [114]. Amplification was performed using DNA isolated from the parental lines of the *L. angustifolius* mapping population, 83A:476 and P27255. Amplicons were extracted directly from the post-reaction mixtures (QIAquick PCR Purification Kit; Qiagen) and sequenced using ABI PRISM 3130 Genetic Analyzer XL (Applied Biosystems, Hitachi, Tokyo, Japan). Allele-specific PCR (AS-PCR) polymorphisms were visualized by 1% agarose gel electrophoresis, whereas nucleotide substitution polymorphisms were revealed by the cleaved amplified polymorphic sequence (CAPS) and dCAPS approaches [115,116]. Restriction sites were identified using dCAPS Finder 2.0 and SNP2dCAPS [117,118]. Digestion products were separated by 2% agarose gel electrophoresis. Multipoint mapping (JoinMap 5, Kyazma, Wageningen, Netherlands) was performed after grouping under independence LOD of 9.5. Some inconsistency in segregation patterns were observed between previously published and newly developed marker sets. In such cases, marker segregation was tested using current DNA isolates (if possible) or questionable data was deleted from segregation file. Linkage group optimization was performed according to the procedure previously applied for white lupin by [54].

### 3.5. Expression Quantitative Trait Loci Mapping

Normalized gene expression values (continuous traits) obtained for RILs and mapping population parental lines were associated with *Ku*, *tardus*, *lentus*, *mollis*, *leucospermus*, *iucundus,* and *Lanr1* segregation data (binary trait) by t-Student test in two classes of polymorphism. Obtained p-values were false discovery rate (FDR) corrected [119]. Genes with corrected *p*-value ≤ 0.01 were subjected to composite interval mapping performed in Windows QTL Cartographer V2.5 (North Carolina State University, Raleigh, NC, USA) using 5 background control markers, window size 10 cM, walk speed 0.5 cM, and backward regression method. LOD threshold for QTL calling was established by permutation test (*N* = 1000) using the same parameters. Linkage groups and LOD graphs were drawn in MapChart [120]. Moreover, sets of genes with corrected *p*-value ≤ 0.01 were analyzed for gene ontology (GO) term enrichment by hypergeometric test with FDR correction in Bingo [121] using GO annotation of *L. angustifolius* genes obtained from Ensembl Plants Genes database (rel. 45, genome assembly LupAngTanjil v1.0). Whole-genome annotation was used as reference set. Results were provided as −log10 (corrected *p*-value).

## 4. Conclusions

1. The massive analysis of cDNA ends was revealed to be applicable for molecular marker development and linkage map construction, as well as for gene expression evaluation and expression quantitative trait loci mapping.
2. The analysis of vernalization independence *Ku* locus shed light on vernalization response via *FLOWERING LOCUS T* and *FD* regulon, providing transcriptomic evidence for contribution of several genes acting in C-repeat binding factor (*CBF*) cold responsiveness and in *UDP*-glycosyltransferases pathways. This information can be relevant to decipher vernalization pathway in legumes, because legume genomes do not contain a major vernalization-responsive gene *FLOWERING LOCUS C* (*FLC*) but other genes from this pathway, including activators and repressors of *FLC*, are present.
3. The study of low-alkaloid *iucundus* locus highlighted a high number of *cis*- and *trans*-regulated alkaloid biosynthesis genes with gene expression orchestrated by a regulatory agent localized at *iucundus* locus, supporting the concept that the *ETHYLENE RESPONSIVE TRANSCRIPTION FACTOR RAP2-7* gene may control low-alkaloid phenotype in narrow-leafed lupin.
4. Research on reduced pod shattering *lentus* locus selected a *DUF1218* domain homolog as a candidate gene controlling the orientation of the sclerified endocarp and a *DETOXIFICATION14* homolog for purplish hue of young pods.
5. An *ABCG* transporter gene was identified as a hypothetical contributor to sclerenchyma fortification underlying reduced pod shattering *tardus* locus.

## Figures and Tables

**Table 1:** Characteristics of the MACE-based *L. angustifolius* linkage map.

Linkage Group	Number of Markers	Number of Loci	Number of Genes	Length (cM)	Number of Scaffolds
NLL-01	289	120	251	156.38	11
NLL-02	207	73	185	132.78	21
NLL-03	204	77	169	115.02	22
NLL-04	193	95	157	126.93	8
NLL-05	144	70	130	101.29	10
NLL-06	304	119	267	144.66	10
NLL-07	191	85	155	104.25	10
NLL-08	231	101	194	119.29	8
NLL-09	192	93	169	140.98	10
NLL-10	154	75	121	85.64	6
NLL-11	269	107	216	119.57	8
NLL-12	271	88	220	85.86	15
NLL-13	230	81	200	78.5	11
NLL-14	160	76	121	87.5	10
NLL-15	248	87	207	86.65	10
NLL-16	240	96	204	99.42	9
NLL-17	227	70	176	91.57	6
NLL-18	196	77	151	91.34	9
NLL-19	144	60	122	92.08	10
NLL-20	199	85	169	103.92	9
Total	4309	1735	3590	2163.63	209

**Table 2:** Expression quantitative trait loci localized near (≤2 cM) major domestication trait loci.

Domestication Trait	Genes with eQTL ^1^ Peak	Mean eQTL Peak LOD Value	Maximum eQTL Peak LOD Value	Genes with *cis* Genomic Positions	Genes with *trans* Genomic Positions	Genes in Unassigned Scaffolds
*iucundus*	61	14.77	37.70	11	45	5
*Ku*	25	11.20	40.97	11	11	3
*leucospermus*	9	13.18	35.34	5	3	1
*lentus*	6	16.36	42.97	5	1	0
*tardus*	2	25.05	31.82	2	0	0
*Lanr1*	4	10.20	13.48	4	0	0
*mollis*	1	9.59	9.59	0	1	0

^1^ eQTL, expression quantitative trait locus.

**Table 3:** Genes showing the highest gene expression association and eQTL peak co-localization (≤2 cM) with low-alkaloid *iucundus* locus.

Protein	Group	Peak cM	Peak LOD	PVE ^1^ %	Association Based on *t*-Student Test	Protein Annotation
OIW21347.1	NLL-07	40.2	37.7	84.2	−0.82	voltage-gated potassium channel subunit beta 1, KAB1
OIV96299.1	NLL-07	40.1	37.3	73.4	0.81	lysine/ornithine decarboxylase, LDC
OIV96574.1	NLL-07	40.1	35.4	60.5	0.84	purine permease 1, PUP1
OIW10551.1	NLL-07	39.6	29.4	67.2	0.80	MLP-like protein 423, MLP423
OIV89004.1	NLL-07	39.6	29.2	56.5	0.78	ethylene-responsive transcription factor, RAP2-7
OIW02927.1	NLL-07	39.6	27.3	69.9	0.69	amino acid permease, AAP
OIW07732.1	NLL-07	41.3	26.3	81.9	−0.73	uncharacterized protein
OIW13431.1	NLL-07	40.2	25.9	59.3	0.78	dihydroflavonol 4-reductase, DFR
OIV89008.1	NLL-07	40.2	25.8	66.2	−0.77	Fe superoxide dismutase 2, FSD2
OIV90042.1	NLL-07	39.6	24.6	59.6	0.72	aspartate kinase 1, AK1
OIV95196.1	NLL-07	39.6	24.3	57.2	0.77	HXXXD-type ACYL-TRANSFERASE, LaAT
OIW13432.1	NLL-07	40.1	24.0	50.2	0.77	dihydroflavonol 4-reductase, DFR
OIW03412.1	NLL-07	40.1	23.5	50.7	0.75	homeobox protein knotted-1-like, KNAT1
OIW15620.1	NLL-07	40.2	22.7	56.6	0.67	uncharacterized protein
OIW20548.1	NLL-07	40.1	21.7	50.3	0.72	diaminopimelate decarboxylase 1, DAPDC1
OIW02909.1	NLL-07	40.1	20.4	53.3	0.72	cytochrome P450, CYP71B23
OIV89669.1	NLL-07	39.6	20.4	48.0	0.70	cinnamoyl-CoA reductase 1, CCR1
OIV96948.1	NLL-07	40.1	19.5	38.5	0.73	copper amine oxidase, MHK10.21
OIW20507.1	NLL-07	40.1	18.3	40.1	0.70	anthocyanidin reductase, BAN
OIW20661.1	NLL-07	41.3	18.0	49.4	0.70	4-hydroxy-tetrahydrodipicolinate synthase, DHDPS
OIV97872.1	NLL-07	39.6	17.2	43.5	0.72	purine permease 1, PUP1
OIV93156.1	NLL-07	40.8	14.9	45.1	0.64	LL-diaminopimelate aminotransferase, DapL
OIV97100.1	NLL-07	39.6	13.9	44.4	0.64	MYB transcription factor 34, MYB34
OIW07643.1	NLL-07	39.0	13.7	36.3	0.64	4-hydroxy-tetrahydrodipicolinate synthase, DHDPS
OIV89772.1	NLL-07	39.6	13.3	40.7	0.51	pentatricopeptide repeat-containing protein
OIW10549.1	NLL-07	40.2	12.7	38.4	0.57	MLP-like protein 423, MLP423
OIV96820.1	NLL-07	40.2	11.8	30.5	0.57	glutamate synthase 1, GLT1
OIW21355.1	NLL-07	39.6	11.8	32.3	0.56	carboxylesterase 1, CXE1
OIW10098.1	NLL-07	40.1	11.8	28.3	0.53	aspartate-semialdehyde dehydrogenase, ASDH
OIW04462.1	NLL-07	39.6	11.7	30.8	0.57	VQ motif-containing protein
OIW10550.1	NLL-07	40.1	10.2	14.9	0.74	MLP-like protein 423, MLP423
OIW20088.1	NLL-07	39.6	9.6	27.4	0.53	short-chain dehydrogenase reductase, SDR
OIV89148.1	NLL-07	41.3	8.2	22.4	0.51	MLP-like protein 31, MLP31
OIW02362.1	NLL-07	39.6	7.9	21.4	−0.55	DMR6-like OXYGENASE 2, 2OG

^1^ PVE, proportion of explained variance.

**Table 4:** Genes showing the highest gene expression association and eQTL peak LOD co-localization (≤2 cM) with vernalization independence *Ku* locus.

Protein	Group	Peak cM	Peak LOD	PVE ^1^ %	Association Based on *t*-Student Test	Protein Annotation
OIW03171.1	NLL-10	50.8	41.0	92.1	0.85	calcium/calmodulin-regulated receptor-like kinase 1, CRLK1
OIW20567.1	NLL-10	49.7	38.9	65.1	−0.89	uncharacterized protein
OIV96743.1	NLL-10	51.4	18.4	53.5	−0.71	galacturonosyltransferase 10-like, GAUT10
OIW03144.1	NLL-10	51.3	16.7	45.2	0.62	MHM17-10, AT5G56980
OIW03199.1	NLL-10	48.4	16.4	50.5	0.69	general transcription factor IIH subunit 2, GTF2H2
OIW03193.1	NLL-10	50.3	10.6	31.5	−0.53	pentatricopeptide repeat-containing protein
OIW20134.1	NLL-10	49.7	9.7	28.9	0.52	UDP-Glycosyltransferase 85A2, UGT85A2
OIV89838.1	NLL-10	51.4	9.6	30.6	0.51	reticulon family protein
OIW19675.1	NLL-10	50.3	8.9	22.6	−0.56	MADS-box transcription factor AGAMOUS-LIKE 8, AGL8
OIW03269.1	NLL-10	52.0	8.5	24.6	0.51	MYB transcription factor 60, MYB60
OIV92673.1	NLL-10	50.3	7.4	22.2	0.53	protein FD-like, FD
OIW03334.1	NLL-10	49.1	7.0	26.7	−0.51	flowering locus protein T, LanFTc1

^1^ PVE, proportion of explained variance.

**Table 5:** Genes showing the highest gene expression association and eQTL peak LOD co-localization (≤2 cM) with anthracnose resistance *Lanr1*, white flower color *leucospermus*, soft seededness *mollis,* and pod shattering *tardus* and *lentus* loci.

Trait	Protein	Group	Peak cM	Peak LOD	PVE ^1^ %	Association Based on *t*-Student Test	Protein Annotation
*Lanr1*	OIW02433.1	NLL-11	41.7	13.5	38.0	0.57	adenine nucleotide alpha hydrolases-like
*Lanr1*	OIW02411.1	NLL-11	41.6	9.0	29.0	−0.51	galactosyltransferase family protein
*le*	OIW06948.1	NLL-08	75.2	43.0	75.8	0.76	fiber protein Fb34, DUF1218
*le*	OIW06960.1	NLL-08	73.3	17.4	45.8	−0.66	MATE efflux family protein DETOXIFICATION14, DTX14
*le*	OIW06846.1	NLL-08	75.2	13.0	38.6	0.56	CDP-diacylglycerol–glycerol-3-phosphate 3-phosphatidyltransferase 2, PGPS2
*leuc*	OIW21684.1	NLL-03	59.1	35.3	75.1	−0.86	ubiquitin-60S ribosomal protein L40A, RPL40A
*leuc*	OIW15321.1	NLL-03	59.6	20.8	53.4	0.76	nascent polypeptide-associated complex subunit alpha 2, NACA2
*leuc*	OIW15287.1	NLL-03	59.1	14.7	40.4	0.59	F-box/WD repeat-containing protein
*leuc*	OIV97389.1	NLL-03	56.1	13.1	36.6	−0.54	protein NRT1/ PTR FAMILY 3.1-like, NPF3.1
*leuc*	OIV89020.1	NLL-03	59.1	10.4	28.6	0.57	GATA type zinc finger transcription factor, WLIM2a
*mol*	OIW15058.1	NLL-17	79.0	9.6	34.6	−0.50	FAM32A, 7-dehydrocholesterol reductase, DWARF5
*ta*	OIW17837.1	NLL-01	34.3	31.8	70.9	−0.79	BolA-like family protein 2, BolA2
*ta*	OIW17820.1	NLL-01	33.1	18.3	44.6	0.66	G-family ATP-binding ABC transporter 5, ABCG5

^1^ PVE, proportion of explained variance.

## Supplementary Materials

Supplementary materials can be found at https://www.mdpi.com/1422-0067/20/22/5670/s1.

## Abbreviations

2OG	DMR6-like OXYGENASE 2
AAP	Amino acid permease
ABCG5	G-family ATP-binding ABC transporter 5
AGL8	AGAMOUS-LIKE 8
AK1	Aspartate kinase 1
AP1	APETALA1
ASDH	Aspartate-semialdehyde dehydrogenase
BAC	Bacterial artificial chromosome
BAN	Anthocyanidin reductase
BolA2	BolA-like family protein 2
CBF	C-repeat binding factor
CCR1	Cinnamoyl-CoA reductase 1
CRLK1	CALCIUM/CALMODULIN-REGULATED RECEPTOR-LIKE KINASE 1
CXE1	Carboxylesterase 1
DAPDC1	Diaminopimelate decarboxylase 1
DapL	LL-diaminopimelate aminotransferase
DArT	Diversity Arrays Technology
DFR	Dihydroflavonol 4-reductase
DHDPS	4-Hydroxy-tetrahydrodipicolinate synthase
DTX14	MATE efflux family protein DETOXIFICATION14
DUF1218	Fiber protein Fb34, domain of unknown function 1218
eQTL	Expression quantitative trait locus
FKF1	FLAVIN-BINDING KELCH REPEAT, F-BOX 1
FLC	FLOWERING LOCUS C
FSD2	Fe superoxide dismutase 2
FT	FLOWERING LOCUS T
FUL	FRUITFULL
GAUT10	Galacturonosyltransferase 10-like
GLT1	Glutamate synthase 1
GO	GENE Gene ontology
GTF2H2	General transcription factor IIH subunit 2
GWAS	Genome-wide association study
ICE1	INDUCER OF CBF EXPRESSION 1
KAB1	Voltage-gated potassium channel subunit beta 1
KNAT1	Homeobox protein knotted-1-like
LaAT	HXXXD-type ACYL-TRANSFERASE
LanFTc1	*L. angustifolius* FLOWERING LOCUS T c1
LDC	Lysine/ornithine decarboxylase
LFY	LEAFY
MACE	Massive analysis of cDNA ends
MATE	Multidrug and toxic compound extrusion
MFLP	Molecular fragment length polymorphism
MHK10.21	Copper amine oxidase
MLP31	MAJOR LATEX PROTEIN 31
MLP423	MAJOR LATEX PROTEIN 423
MWL-1	MODIFYING WALL LIGNIN-1
MWL-2	MODIFYING WALL LIGNIN-2
MYB60	MYB transcription factor 60
NACA2	Nascent polypeptide-associated complex subunit alpha 2
NPF3.1	Protein NRT1/ PTR FAMILY 3.1-like
PGPS2	CDP-diacylglycerol–glycerol-3-phosphate 3-phosphatidyltransferase 2
PUP	Purine permease transporter
RAP2-7	ETHYLENE RESPONSIVE TRANSCRIPTION FACTOR RAP2-7
RIL	Recombinant inbred line
RPL40A	Ubiquitin-60S ribosomal protein L40A
SDR	Short-chain dehydrogenase reductase
SNP	Single nucleotide polymorphism
SOC1	SUPPRESSOR OF OVEREXPRESSION OF CONSTANS 1
UGT85A2	UDP-Glycosyltransferase 85A2
UGT87A2	UDP-Glycosyltransferase 87A2
